# Sex-Stratified Correlates of Pulmonary Function in Mexican Children and Adolescents (6–17 Years) with Asthma: An Exploratory Analysis of HDL Cholesterol, BMI, and Pubertal Stage

**DOI:** 10.3390/nu18121885

**Published:** 2026-06-11

**Authors:** Nayely Reyes-Noriega, José J. Leija-Martínez, Fausto Sánchez-Muñoz, Adrián Hernández-Díazcouder, Claudia Tavera Alonso, Santiago Villafaña, Darío Jorge Mario Molina Díaz, Blanca E. Del-Río-Navarro, Fengyang Huang

**Affiliations:** 1 Laboratorio de Investigación en Alergia e Inmunología, Hospital Infantil de México Federico Gómez, Dr Márquez 162, Cuauhtémoc, Mexico City 06720, Mexico; naye.rey.nor@gmail.com; 2 Hospital Regional de Alta Especialidad “Dr. Ignacio Morones Prieto”, IMSS-Bienestar, Av. Venustiano Carranza No. 2395, San Luis Potosí 78290, Mexico; leijamjj@gmail.com; 3 Departamento de Fisiología, Instituto Nacional de Cardiología Ignacio Chávez, Mexico City 14080, Mexico; fausto22@yahoo.com; 4 Unidad de Investigación Médica en Bioquímica, Hospital de Especialidades, Centro Médico Nacional Siglo XXI, Instituto Mexicano del Seguro Social, Mexico City 06720, Mexico; adrian.hernandez.diazc@gmail.com; 5 Laboratorio Central, Instituto Nacional de Cardiología Ignacio Chávez, Mexico City 14080, Mexico; taveramuc@yahoo.com.mx; 6 Laboratorio de Terapia Génica Experimental, Escuela Superior de Medicina, Instituto Politécnico Nacional, Mexico City 11340, Mexico; svillafana@ipn.mx; 7 Departamento de Endocrinología, Hospital Infantil de México Federico Gómez, Dr Márquez 162, Cuauhtémoc, Mexico City 06720, Mexico; drjorgemariomolina@gmail.com; 8 Laboratorio de Investigación en Obesidad y Asma, Hospital Infantil de México Federico Gómez, Mexico City 06720, Mexico

**Keywords:** asthma, children, adolescents, pubertal development, FEV1, FVC, HDL cholesterol, sex differences, asthma control

## Abstract

**Background/Objectives:** Pubertal maturation encompasses hormonal, somatic, and metabolic alterations that may influence pulmonary function in children with asthma. We evaluated the correlates of pulmonary function in pediatric asthma using sex-stratified analyses of pubertal stage, BMI z-score, asthma control (ACQ-6), and HDL cholesterol levels on FEV1, FVC, and the FEV1/FVC ratio (% predicted, NHANES III). **Methods:** A cross-sectional study was conducted with 118 children and adolescents with asthma (74 males and 44 females). The Tanner stages were categorized as pre-/early (I–II), mid (III), and late (IV). No participant had reached Tanner stage V at recruitment, and the reference category was composed predominantly of Tanner II participants; therefore, the pubertal contrast reflected early- versus mid-/late-pubertal physiology rather than a strictly prepubertal-versus-pubertal comparison. We performed hierarchical linear regression with bias-corrected and accelerated bootstrap inference (5000 replicates) for each sex and for all participants combined, complemented by a formal sex × predictor interaction test. **Results:** Mid- and late puberty were independently associated with reduced FEV1% and FVC% (*β* −0.25 to −0.31), without affecting the FEV1/FVC ratio. Sex-stratified analyses were exploratory and identified apparently divergent predictor sets by sex: in males, the pubertal stage was significant in FEV1% and FVC% (*β* −0.30 to −0.42), whereas the BMI z-score diminished the FEV1/FVC ratio (*β* = −0.274). In females, FEV1% was associated with HDL-c (*β* = 0.463), BMI z-score (*β* = 0.429), and ACQ-6 score (*β* = −0.306); the female FEV1/FVC% model showed a substantial apparent effect (Cohen’s f^2^ = 0.508), with ACQ-6 score and HDL-c as primary associated variables. The formal sex × predictor interaction test in the combined sample, however, did not reach statistical significance (Δ*R*^2^ = 0.037; *p* = 0.178), indicating that the sex-differential patterns observed in the stratified models were not confirmed as effect modifications. **Conclusions:** Sex-stratified exploratory analyses in this pediatric asthma cohort identified apparently divergent patterns of associations in males and females; however, the formal interaction test was not statistically significant, indicating that these stratified differences were not confirmed and require validation in adequately powered prospective cohorts. These findings identify HDL-c, body composition, and symptom control as candidate correlates of pulmonary function, with potential sex-related differences warranting further investigation.

## 1. Introduction

Asthma is a common chronic respiratory disease that affects approximately 14% of children and adolescents worldwide. This disease negatively impacts their health, resulting in school absenteeism, frequent medical attention, and diminished quality of life [1]. According to the Global Asthma Network Study in Mexico City, the number of children experiencing severe asthma symptoms has increased over the last decade, particularly during challenging adolescent years [2]. During puberty, changes occur in relation to asthma, representing a critical period for disease modulation [3,4], which is reflected in sex-specific phenotypes, severity, and control of the disease [5,6]. During childhood, asthma is more prevalent and severe in males than in females. However, once they reach adolescence, the situation reverses, and females begin to encounter more asthma symptoms, greater airway hyperresponsiveness, and worse lung function trajectories [7,8]. This transition, often referred to as the “gender switch” in asthma epidemiology [9], frequently correlates with elevated sex steroid levels, body composition, and metabolic and immune function [10,11].

### 1.1. Pulmonary Function During Pubertal Growth

Throughout childhood and adolescence, respiratory system development continues, with non-uniform growth velocities in distinct anatomical compartments [12,13]. The lung parenchyma and thoracic dimensions endure rapid expansion during the pubertal growth spurt, particularly in males, whereas airway caliber maturation follows a partially independent trajectory, a phenomenon termed dysanaptic growth [14]. This results in a distinctive functional pattern of relatively preserved or increased forced vital capacity (FVC) and total lung capacity (TLC), with reduced maximal expiratory flows and a reduced FEV1/FVC ratio [15,16]. Pediatric research has demonstrated that dysanaptic growth is independently linked to both obesity and asthma [15]. In addition, an accelerated weight growth velocity during infancy and puberty has been correlated with disproportionate lung size and airway development, an elevated risk of wheezing and asthma, and a diminished FEV1/FVC ratio [17]. Moreover, longitudinal evidence indicates that pubertal height velocity is distinctly correlated with FEV1 trajectories in adolescents and young adults [18], which has direct implications for interpreting spirometry reference values throughout puberty [19,20].

### 1.2. Sex Dimorphism in Asthma: Genetic, Hormonal and Immune Substrates

The biological basis for sex disparities in adolescent asthma involves genetic, epigenetic, hormonal, and metabolic mechanisms in females. Endogenous sex hormones have divergent effects on pulmonary function and the clinical course of asthma. In males, increased levels of testosterone and dehydroepiandrosterone sulfate (DHEA-S) are associated with improved lung function and asthma symptom control. Conversely, in females, advanced pubertal development characterized by a high estrogenic environment induces mast cell degranulation [21], heightens allergic sensitization, and exacerbates bronchial inflammation, leading to poorer outcomes [22,23]. While these mechanisms explain the differential expression of the disease between sexes, some studies have revealed a similar risk of asthma in populations experiencing early puberty [24]. This discrepancy suggests that sex differences extend beyond hormonal variations to include genetic differences in gene regulation [25]. Indeed, asthma is increasingly acknowledged to exhibit sex-related differences at both the genetic and epigenetic levels, including sex-specific patterns of gene expression and DNA methylation that may contribute to the divergent course observed between males and females across puberty [25,26]. In females, a specific haplotype in the promoter region of defensin-β1 (DEFB1) has been linked to a reduced risk of asthma, although this association does not persist into adulthood [27]. Furthermore, the X chromosome contains numerous genes related to immune function, potentially influencing the prevalence and severity of asthma. Although one X chromosome is typically inactivated in females, research suggests that certain immunological mediators involved in the innate immune response, such as Toll-like receptors (TLRs), including TLR7 and TLR8, may escape this X-linked inactivation. Consequently, these receptors may be overexpressed in mononuclear cells in females, contributing to an increased risk of developing inflammatory diseases [28].

At the same time, puberty changes body composition, lipid profiles, and inflammatory marker concentrations [29,30]. These metabolic changes are central to obesity-associated asthma, a recognized phenotype in both adults and children, characterized by excess adiposity, a non-type-2 inflammatory profile, and reduced responsiveness to inhaled corticosteroids. Adipose tissue in this phenotype behaves as an active endocrine and immune organ, and the immunometabolic links between adiposity, systemic low-grade inflammation, and airway disease are increasingly regarded as contributors to its etiopathogenesis [31,32]. Childhood obesity has been linked to an unanticipated increase in FEV1 and FVC, accompanied by a diminished FEV1/FVC ratio, attributed to adiposity-induced dysanapsis [15,33]. Additionally, sex-specific patterns of adipose distribution may yield divergent functional outcomes in males and females [34]. In this context, high-density lipoprotein cholesterol (HDL-c) is a significant metabolic biomarker because of its role in reverse cholesterol transport and its anti-inflammatory and antioxidative properties, facilitated by apolipoprotein A-I and paraoxonase-1 [35,36]. Beyond their cardiometabolic role, HDL particles have been linked to asthma and allergic diseases more broadly; epidemiological and mechanistic studies have reported associations between HDL-c and prevalence, severity, and asthma control, supporting a plausible immunomodulatory connection between lipoprotein metabolism and airway disease [37,38]. These mechanisms are particularly relevant during puberty, given the recognized metabolic alterations in children with asthma [39], along with estrogen-induced enhancement of HDL particle size and ApoA-I synthesis [40]. Interestingly, a previous study on adolescents reported that HDL-c was negatively associated with FEV1 and FVC in healthy male Korean adolescents, suggesting a sex-differential pattern in the HDL-c pulmonary function relationship that has not yet been characterized in pediatric asthma populations [41]. Despite this convergent biological explanation, the potential sex-specificity of HDL–pulmonary function associations in pediatric asthma remains unexplored. Other significant biomarkers closely related to diet, such as 25-hydroxyvitamin D and serum uric acid, may affect airway inflammation through distinct immunomodulatory pathways [42,43,44,45,46,47]. However, their interpretation necessitates meticulous consideration of developmental stage and sex.

In this context, this study aimed to explore this gap by evaluating the independent and sex-specific influences of pubertal stage, body mass index z-score, asthma symptom control, and serum HDL cholesterol on pulmonary function (FEV1% predicted, FVC% predicted, and FEV1/FVC% predicted) in children and adolescents with asthma.

## 2. Materials and Methods

### 2.1. Study Design and Participants

This cross-sectional study was performed at the Allergy and Immunology Research Laboratory of the Hospital Infantil de México Federico Gómez from January 2022 to December 2024, after protocol approval in November 2021 by the Biosafety, Ethics, and Research Committees of the same institution under registration number HIM/2021/023 SSA 1748.

Mexican patients aged 6–17 years with asthma, as defined by the 2022 Global Initiative for Asthma (GINA) standards [1], confirmed by an allergy and immunology specialist, and with a minimum disease duration of 6 months, were recruited.

Before any procedure, the patients and their parents or guardians signed informed consent and assent documents explaining the study’s objectives and methods.

Patients with other pulmonary disorders, such as cystic fibrosis, hepatic, renal, or osseous diseases, hyperthyroidism, hypothyroidism, or mandibular or cranial abnormalities were excluded.

### 2.2. Anthropometric and Clinical Assessment

A board-certified pediatric specialist obtained the medical history and performed a complete physical examination in the presence of a parent or guardian. Data were collected regarding the duration of asthma diagnosis, number of asthma exacerbations in the last month, and the current pharmacological treatment sustained for a minimum of three months in accordance with the GINA stepwise approach [1]. All participants had a diagnosis of allergic asthma. During the standard diagnostic procedure to identify type 2 inflammatory phenotype, fractional exhaled nitric oxide (FeNO) levels were measured using the NIOX VERO^®^ device (NIOX Group plc, Oxford, UK) at an expiratory flow rate of 50 mL/s in accordance with ATS/ERS recommendations [48]. Additionally, skin prick tests were conducted using a standardized set of common aeroallergen extracts (IPI ASAC México, S.A. de C.V., Mexico City, Mexico), such as house dust mites, pollen mixtures, and animal dander, with histamine and saline serving as positive and negative controls, respectively. A wheal diameter ≥3 mm larger than the negative control was considered a positive result [49].

Trained medical professionals followed the standardized procedures described in the Anthropometric Standardization Reference Manual [50] to obtain anthropometric measurements. Body weight was measured to the nearest 0.1 kg using a calibrated digital scale (model 804, capacity 150 kg; SECA GmbH & Co. KG, Hamburg, Germany), while height was measured to the nearest 0.1 cm using a portable stadiometer (model 213, capacity 205 cm; SECA GmbH & Co. KG, Hamburg, Germany). The participants were barefoot, wore light clothing, and were positioned in the Frankfort horizontal plane. Using a non-elastic measuring tape, we measured the waist and hip circumferences to the nearest 0.1 cm at the midpoint between the lowest rib and the iliac crest and at the level of the greater trochanter. The same observer took all measurements twice, and the average of the two readings was used for analysis. If the two measurements differed from the range tolerance described in the reference manual (>0.5 kg for weight, >0.5 cm for height, and >1.0 cm for circumference), a third measurement was performed, and the median value was recorded. Body mass index (BMI) was calculated by dividing weight (kg) by the square of height (m^2^). Age- and sex-specific BMI z-scores were calculated using the 2007 World Health Organization (WHO) growth reference for school-aged children and adolescents [51] and were computed using WHO AnthroPlus software (version 1.0.4; World Health Organization, Geneva, Switzerland). Participants were classified based on their z-score cut-offs as normal weight (−2 ≤ z ≤ +1), overweight (+1 < z ≤ +2), or obesity (z > +2).

Pubertal development was evaluated through direct physical examination using the Tanner staging system [52,53,54,55], the most widely recognized clinical scale for assessing sexual maturation. Participants were categorized into Tanner stages I–V based on breast development and pubic hair in females and genital (testicular and penile) development and pubic hair in males. Consistently, standards were mandated between the two anatomical regions, and in the few instances of discordance, the superior of the two stages was selected.

Asthma control was evaluated using the 6-item Spanish version of the Asthma Control Questionnaire (ACQ-6) [56,57], which was validated by Picado et al. in a multicenter study involving adults with asthma in Spain [58]. We acknowledge the absence of pediatric-specific validation of the ACQ-6 in Spanish; however, linguistic and conceptual equivalence, coupled with the clear wording of the six items, supports its utilization in Spanish-speaking pediatric patients. The ACQ-6 evaluates six domains from the prior week, namely nocturnal awakening, morning symptoms, activity limitation, dyspnea, wheezing, and short-acting agonist utilization, with each item rated on a 7-point scale (0 = no impairment to 6 = maximum impairment). The overall score was the arithmetic mean of the six items, ranging from 0 (optimal control) to 6 (poor control). Scores below 0.75 indicated well-controlled asthma, whereas scores of 1.5 or higher indicated uncontrolled asthma. Scores between 0.75 and 1.5 are regarded as the “grey zone”, which is usually considered as partial control in clinical practice. In the present study, the ACQ-6 was used as one of several indicators of current symptom burden rather than as a standalone diagnostic measure of asthma control, and its scores were therefore interpreted as approximate, exploratory indicators of recent symptom control in the pediatric sample. The lack of formal pediatric Spanish-language validation is explicitly addressed as a limitation in Section 4.8.

### 2.3. Spirometry

Spirometry was performed by a physician certified in spirometry testing through a course approved by The U.S. National Institute for Occupational Safety and Health (NIOSH) utilizing a Vitalograph Pneumotrac model 6800 desktop spirometer (Vitalograph Ltd., Buckingham, UK) integrated with Spirotrac^®^ 6 software. A 3 L precision syringe was used for daily calibration following the manufacturer’s guidelines and the 2019 American Thoracic Society/European Respiratory Society (ATS/ERS) technical standards for spirometry [59]. A new bacterial/viral filter (Vitalograph BVF^TM^; (Vitalograph Ltd., Buckingham, UK).) was used for each participant to prevent cross-contamination.

The ATS/ERS guidelines were used to measure forced vital capacity (FVC), forced expiratory volume in one second (FEV1), and the FEV1/FVC ratio. Each patient performed at least three acceptable maneuvers that satisfied the repeatability criteria (variability ≤ 150 mL or ≤10% between the two best maneuvers). Predicted values and percent-predicted (% predicted) were calculated using the NHANES III reference equations by Hankinson et al. [60], utilizing the Mexican-American sex-specific coefficients due to the patients’ ethnic composition.

After baseline (pre-bronchodilator) spirometry, all participants received 400 µg of inhaled salbutamol via a metered dose inhaler with a valved holding chamber. Post-bronchodilator spirometry was performed 15 min later in accordance with the standard clinical protocol.

The primary outcomes in all multivariable analyses were pre-bronchodilator values of FEV1% predicted, FVC% predicted, and FEV1/FVC% ratio, as they represent the airway resting structural and functional phenotypes.

### 2.4. Biochemical Assessment

All biochemical assays were performed on serum samples obtained from a single morning venous blood draw after an overnight fast (≥8 h). Routine clinical chemistry was performed at the Clinical Laboratory of the Hospital Infantil de México Federico Gómez (Mexico City), and total 25-hydroxyvitamin D levels were evaluated at the Central Laboratory of the Instituto Nacional de Cardiología Ignacio Chávez (Mexico City). Internal and external quality control measures were completed during the study using reagents from a single lot to reduce between-run variability.

#### 2.4.1. Serum Biochemistry: Lipid Profile and Uric Acid

Serum total cholesterol, high-density lipoprotein cholesterol (HDL-c), low-density lipoprotein cholesterol (LDL-c), triglycerides, and uric acid levels were measured on a Beckman Coulter AU480 clinical chemistry analyzer (Beckman Coulter Inc., Brea, CA, USA) using the manufacturer’s reagents, each according to its specific enzymatic method. The catalog references were as follows: total cholesterol (OSR6116, cholesterol oxidase–peroxidase method), HDL cholesterol (OSR6195, direct enzymatic method utilizing detergent-mediated selective solubilization), LDL cholesterol (OSR6196, direct homogeneous enzymatic method), triglycerides (OSR60118, glycerol phosphate oxidase method), and uric acid (OSR6098, uricase–peroxidase method). All assays were calibrated and conducted in accordance with the manufacturer’s package inserts, incorporating two daily level controls at clinically pertinent concentrations.

#### 2.4.2. 25-Hydroxyvitamin D

Total serum 25-hydroxyvitamin D 25(OH)D, comprising 25(OH)D_3_ and 25(OH)D_2_, was quantified using the Elecsys^®^ Vitamin D total II electrochemiluminescence immunoassay (ECLIA; reagent catalog reference 07028148190; calibrator CalSet 07464240190; Roche Diagnostics GmbH, Mannheim, Germany). The assay employs a recombinant ruthenium-labeled vitamin D binding protein (VDBP) that competitively captures both 25(OH)D_3_ and 25(OH)D_2_, while a specific monoclonal antibody inhibits cross-reactivity to 24,25-dihydroxyvitamin D. The three-step competitive electrochemiluminescence reaction was completed in 27 min, in accordance with the manufacturer’s protocol. Quality control was performed at two clinically significant concentrations using the manufacturer’s controls.

The classification of vitamin D status was based on the benchmarks set forth by the 2011 Endocrine Society Clinical Practice Guideline [61]: deficiency is defined as <20 ng/mL (50 nmol/L), insufficiency as 20–29 ng/mL (50–74 nmol/L), and sufficiency as ≥30 ng/mL (75 nmol/L). This guideline was replaced in 2024 by the revised Endocrine Society Guideline on Vitamin D for disease prevention [62], which transitioned the focus from population screening to context-specific empirical supplementation and did not utilize the previous categorical thresholds. Nonetheless, the 2011 cut-off points continue to be the most widely applied clinical research standard and were preserved for this study to enable direct comparability with the current pediatric asthma literature.

### 2.5. Statistical Analysis

All statistical analyses were conducted utilizing IBM SPSS Statistics (version 29.0; IBM Corp., Armonk, NY, USA). Continuous variables were expressed as mean ± standard deviation (SD) along with median and interquartile range (IQR; P25–P75), according to the Shapiro–Wilk test evaluation for normality. Categorical variables were presented as frequencies and percentages. Between-group comparisons by sex were performed using Welch’s *t*-test when both groups satisfied the normality assumption (Shapiro–Wilk *p* > 0.05) and the Mann–Whitney U test when at least one group did not meet this assumption (Shapiro–Wilk *p* < 0.05). Categorical variables were analyzed using Pearson’s χ^2^ test when all expected cell counts were ≥5 and Fisher’s exact test when at least one expected cell count was <5. Statistical significance was established at *p* < 0.05 (two-tailed).

All participants provided comprehensive data on all study variables, including demographic, anthropometric, clinical, biochemical, and spirometry parameters, which were analyzed to delineate the overarching landscape of pulmonary function (Supplementary Table S1). Consequently, no imputation methods were employed, and the analytic sample sizes represented the total number of participants, except when the sensitivity analysis or model-specific influence diagnostics necessitated the exclusion of influential observations (refer to Section 2.5.4 for further details).

#### 2.5.1. Hierarchical Multiple Linear Regression

**Figure 1:**
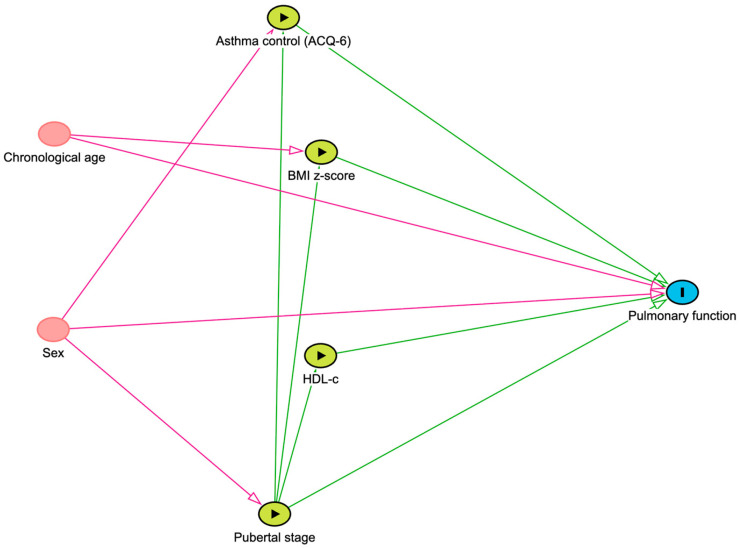
Directed acyclic graph (DAG) representing the assumed a priori causal structure used to guide covariate selection and model specification, constructed using DAGitty (dagitty.net). Biological sex and chronological age are upstream common causes and were adjusted as covariates in the combined-sample models. Pubertal stage, BMI z-score, serum HDL-c, and asthma symptom control (ACQ-6 score) were the exposures of primary interest, each with a hypothesized direct effect on pulmonary function (FEV1%, FVC%, and FEV1/FVC% predicted). The arrows represent theoretical assumptions derived from the prior literature and do not depict causal relationships demonstrated by the present cross-sectional data. Exposures of interest are shown in green, the outcome in blue, and adjusted common causes in pink.

Three simultaneous two-block hierarchical multiple linear regression models were constructed to ascertain the independent predictors of FEV1% predicted, FVC% predicted, and FEV1/FVC% predicted. The predictors entered into the combined-sample models were not selected by data-driven screening but were pre-specified based on an explicit theoretical framework. A directed acyclic graph (Figure 1) was constructed a priori to represent the assumed causal structure linking sex, chronological age, pubertal stage, BMI z-score, serum HDL-c, and asthma symptom control. The assumed causal paths were grounded in the prior literature: the effect of pubertal stage on lung function is supported by studies of pubertal lung and thoracic growth [12,13,18,24]; the influence of pubertal maturation on metabolic and lipid status by evidence of lipid changes across puberty [63]; the effect of adiposity on lung function by studies of obesity and airway dysanapsis [15,33,64]; the association of adiposity with metabolic markers and with asthma in children [3,4,39]; the role of sex hormones and sex-related mechanisms in asthma and lung function [11,22,23]; and the association of pubertal stage with asthma symptom control by evidence of the pubertal shift in asthma expression and the effect of pubertal timing in asthma risk [9,24]. In this framework, biological sex and chronological age are considered upstream common causes and are therefore adjusted as covariates rather than being treated as primary exposures. In contrast, this study aimed to estimate the independent associations of pubertal stage, BMI z-score, HDL-c, and the ACQ-6 score with pulmonary function. A directed acyclic graph was used to intentionally limit the combined model to this parsimonious set of predictors, ensuring an adequate observations-per-parameter ratio (Section 2.5.4) and avoiding over-adjustments. The graph encodes a priori assumptions solely for guiding model specification and covariate selection; it does not represent, nor should it be interpreted as, evidence of causal relationships, which the cross-sectional design of the current study cannot establish.

The block assignment followed a temporal–causal logic rather than a strictly biological one: Block 1 contained variables that were demographically prior to or anatomically antecedent to the clinical, metabolic, and pubertal predictors in Block 2. Sex is fixed at conception, and the BMI z-score (calculated from anthropometric measurements obtained at study entry) reflects long-term cumulative growth that has been operative throughout the participant’s life. Block 2 captured proximal predictors that may causally interact with pulmonary function during the immediate evaluation window: current asthma symptom control (ACQ-6, prior 7 days), current metabolic status (HDL-c level measured at study entry), and current pubertal maturation (Tanner stage at the examination). We acknowledge that the BMI z-score has metabolic significance and could be argued to belong in Block 2; we performed an exploratory re-specification with the BMI z-score in Block 2, and the substantive conclusions did not change. The block ordering reported in this manuscript was therefore retained for parsimony and consistency with established hierarchical regression conventions for pediatric studies [51].

For Tanner stage, we classified the patients into three groups for the regression analysis: pre-/early puberty (Tanner I–II, reference; *n* = 46), mid-puberty (Tanner III; *n* = 35), and late puberty (Tanner IV; *n* = 37). Two dummy variables were created: mid-puberty = 1 if Tanner III, and late puberty = 1 if Tanner IV. No participant in the recruited cohort had reached Tanner V at the time of evaluation, consistent with the upper age limit of 17 years and the predominance of mid-pubertal participants. Within the reference category, Tanner II accounted for the majority of observations (38 of 46; 82.6%), with only eight prepubertal Tanner I participants (6.8% of the total cohort); therefore, the reference category represents predominantly early pubertal rather than strictly prepubertal physiology, and the contrasts estimated by the dummy variables should be interpreted as differences between early- vs. mid- or late-pubertal stages.

#### 2.5.2. Analyses Stratified by Sex and Examination of Sex-by-Predictor Interactions

To evaluate potential sex-specific trends in the predictors of pulmonary function, hierarchical models were analyzed independently for male (*n* = 74) and female (*n* = 44) participants. The sex variable was excluded from the stratified models, and Block 1 included only the BMI z-score. A comprehensive analysis of sex × predictor interactions was conducted on the entire sample to formally evaluate whether the influence of the predictors on pulmonary function differed according to sex. We added interaction terms (sex × mid-puberty, sex × late puberty, and sex × HDL-c) as additional blocks in the combined hierarchical regression model. We then evaluated the resultant change in *R*^2^ (Δ*R*^2^*)* using an *F*-test to determine the increase in the explained variance. To reduce multicollinearity caused by product terms, continuous predictors were mean-centered before creating interaction terms, as is standard practice for moderation analysis [65]. Sex-stratified analyses were preordained and executed regardless of the results of the global interaction test, grounded in the a priori hypothesis of sex-differential effects and recognized constraints in statistical power for detecting interactions in observational studies [66]. Disease severity and treatment are potential confounders of the association between metabolic markers and lung function. Because the GINA treatment step and the ACQ-6 score are strongly correlated and capture overlapping information, entering both in the same model would introduce collinearity; therefore, we selected the ACQ-6 score as the primary control variable, as it provides a finer-grained, week-level measure of current symptom burden. In the pre-specified exploratory analysis, the GINA treatment step did not contribute independent variance to any of the three pulmonary function outcomes once the ACQ-6 score was included (Supplementary Table S1), supporting this specification. The residual potential for confounding by disease severity and inhaled corticosteroid exposure is acknowledged in the Limitations Section (Section 4.8).

#### 2.5.3. Bootstrap BCa Estimation

The Shapiro–Wilk test was used to evaluate the normality of the residuals across all models. Owing to the deviation of residuals from normality in the majority of models, bias-corrected and accelerated (BCa) bootstrap resampling with 5000 replicates was implemented to obtain inferentially valid 95% confidence intervals and significance levels [67,68]. The BCa method corrects for both bias and skewness in bootstrap distributions. Both ordinary least squares (OLS) and bootstrap BCa estimates were derived using the BOOTSTRAP procedure of IBM SPSS Statistics version 29.0 (IBM Corp., Armonk, NY, USA), which implements the BCa interval according to Efron and Tibshirani [66]. Non-converged bootstrap replicates were excluded from the BCa calculation by default; the proportion of non-converged replicates is reported in the Results Section when relevant (Section 3.3.4).

To address the family-wise error inherent in the multiple sex-stratified contrasts (5 predictors × 3 pulmonary outcomes × 2 sexes = 30 simultaneous bootstrap-derived hypothesis tests across Table 3, Table 4 and Table 5), we applied Benjamini–Hochberg false discovery rate (FDR) correction to the family of bootstrap BCa p-values from the sex-stratified analyses. Adjusted p-values (*q*-values) were computed as *q* = min [1, p × m/rank], where m = 30 is the total number of contrasts and rank is the rank of the contrast in ascending order of raw p-values; *q*-values were then enforced to be monotonic in rank to prevent rank-reversal artifacts. We pre-specified two interpretive thresholds: *q* < 0.05 as the primary criterion for retained statistical significance under FDR control and *q* < 0.10 as a secondary “marginal” zone for transparent reporting. The full FDR-corrected results are presented in Supplementary Table S6.

#### 2.5.4. Sensitivity Analyses

Two distinct observations exhibited Cook’s distance exceeding 1 in the regression models. The first observation (Cook’s distance = 6.875) was excluded from the primary analyses of the combined FEV1/FVC% predicted model (*n* = 117), the male sex-stratified FEV1% predicted model (*n* = 73), and the male sex-stratified FEV1/FVC% predicted model (*n* = 73) because its leverage was approximately seven times the threshold, and its removal restored model normality and stabilized the bootstrap distribution. The second observation (Cook’s distance = 3.008) affected the male sex-stratified FVC% predicted model. Although this observation also exceeded the D > 1 threshold, we retained it in the primary FVC% analysis and evaluated its impact through a pre-specified sensitivity analysis (Supplementary Table S2; *n* = 73). The decision to retain rather than exclude this case in the primary model was motivated by two considerations: (i) the bootstrap BCa interval already signaled the instability of the BMI z-score coefficient by crossing zero (see footnote ^b^ in Table 4), allowing readers to detect the dependence on a single observation without further intervention by the analyst, and (ii) excluding both influential cases from the primary analyses would have resulted in inconsistent sample sizes across the male-stratified outcomes. In our view, the transparency of this approach, retaining the case, displaying BCa instability, and explicitly performing the sensitivity analysis are preferable to model-specific exclusion rules that could be perceived as post hoc.

In addition, the sample size of *n* = 118 yielded approximately 14 observations for each estimated parameter in the combined model, consistent with the recommended 10–15 observations per parameter for reliable regression estimation [69]. Because the cohort was fully enrolled before analysis, the sample size was fixed by design rather than derived from an a priori power calculation; the predictor set for the combined model was therefore deliberately restricted to five conceptually motivated variables (sex, BMI z-score, ACQ-6, HDL-c, and pubertal stage) presented in Figure 1. The sex-stratified models were consequently presented as an exploratory, hypothesis-generating extension of the primary combined-sample analysis, and the female-stratified estimates should be interpreted with the caution warranted by a smaller subsample (Section 4.8).

#### 2.5.5. Effect Size and Model Diagnostics

The model fit was evaluated using *R*^2^*,* adjusted *R*^2^*,* standard error of estimate (SEE), and Cohen’s *f*^2^*,* calculated as (*R*^2^_2_ − *R*^2^_1_)/(1 − *R*^2^_2_), where values of 0.02, 0.15, and 0.35 indicate small, medium, and large effects, respectively [70]. Multicollinearity was assessed using variance inflation factors (VIFs), with a VIF threshold of <5 being acceptable (Supplementary Table S4). Multicollinearity was assessed using variance inflation factors (VIFs), with a VIF threshold of <5 being acceptable (Supplementary Table S4). All VIFs were below 1.4, and tolerance values exceeded 0.74 across the three combined-sample models, well within conventional thresholds, indicating the absence of problematic collinearity.

## 3. Results

### 3.1. Participant Characteristics

The study comprised 118 children and adolescents with asthma (74 males and 44 females), with a mean age of 10.6 ± 2.6 years, as detailed in Table 1, alongside other demographic, clinical, and biochemical characteristics of the participants. All participants had allergic asthma, with a high prevalence of aeroallergen sensitization (positive skin prick in 90.7% of the cohort) and a median FeNO level of 22.0 ppb. Neither FeNO nor skin prick test positivity differed significantly between the sexes (*p* = 0.568 and *p* = 0.611, respectively). Two patterns of sex-related divergence were identified from the baseline comparisons: females exhibited greater advancement in pubertal maturation than males (median age 12.3 vs. 10.0 years; Tanner IV: 43.2% vs. 24.3%), presented a more severe asthma phenotype, with a higher prevalence in GINA Steps 4–5 (34.0% vs. 9.5%; *p* = 0.005, Fisher’s exact test), and demonstrated poorer symptom control on the ACQ-6 score (*p* = 0.032). In addition, the female group exhibited significantly lower serum 25-hydroxyvitamin D concentrations (*p* = 0.001), with no female participant meeting the sufficiency criteria (≥30 ng/mL).

Moreover, the prevalence of obesity was higher in males than in females (31.1% vs. 13.6%). Despite these clinical and metabolic differences, lung function parameters (FEV1%, FVC%, and FEV1/FVC%) did not differ significantly between the sexes (all *p* > 0.5).

**Table 1:** Demographic, anthropometric, clinical, and biochemical characteristics of the study patients by sex.

Variables	Total *n* = 118	Males *n* = 74	Females *n* = 44	*p*-Value
**Demographic and anthropometric**				
Age (years)	10.6 ± 2.6 (10.8 [8.32–13.1])	10.2 ± 2.6 (10.0 [7.7–12.3])	11.2 ± 2.5 (12.3 [8.5–13.4])	0.064 (MW)
Height (cm)	142.3 ± 16.1 (143.8 [128.9–155.6])	140.9 ± 16.9 (139.8 [127.4–156.4])	144.6 ± 14.5 (150.6 [130.5–155.2])	0.087 (MW)
Weight (kg)	43.7 ± 16.8 (43.7 [28.7–53.2])	42.9 ± 17.7 (42.1 [27.6–53.2])	45.0 ± 15.2 (46.0 [30.7–54.7])	0.498 (t)
BMI (kg/m^2^)	20.9 ± 5.0 (20.1 [17.1–23.4])	21.0 ± 5.6 (19.6 [16.7–23.4])	20.8 ± 3.9 (20.4 [18.0–23.4])	0.341 (MW)
BMI z-score	1.16 ± 1.28 (1.04 [0.19–1.98])	1.2 ± 1.40 (1.32 [0.11–2.25])	0.96 ± 0.90 (0.85 [0.30–1.55])	0.272 (MW)
BMI category, *n* (%)				
Normal weight	61 (51.7)	37 (50.0)	24 (54.5)	0.067 (FE)
Overweight	28 (23.7)	14 (18.9)	14 (31.8)	
Obesity	29 (24.6)	23 (31.1)	6 (13.6)	
Waist circumference	75.6 ± 13.2 (74.0 [65.0–82.6])	75.6 ± 14.4 (74.5 [62.3–84.2])	74.3 ± 11.0 (73.2 [67.0–81.8])	0.504 (MW)
Hip circumference	77.9 ± 12.3 (78.5 [67.0–85.5])	76.6 ± 12.8 (77.0 [66.7–85.1])	80.1 ± 11.4 (81.7 [71.0–88.2])	0.135 (t)
**Pubertal stage (Tanner)**				
Tanner stage, *n* (%)				
I	8 (6.8)	6 (8.1)	2 (4.5)	
II	38 (32.3)	27 (36.5)	11 (25.0)	
I–II (combined for analysis)	46 (39.0)	33 (44.6)	13 (29.5)	0.065 (χ^2^)
III	35 (29.7)	23 (31.1)	12 (27.3)	
IV	37 (31.4)	18 (24.3)	19 (43.2)	
V	0 (0.0)	0 (0.0)	0 (0.0)	
**Asthma phenotype, severity, and control**				
Allergic asthma, *n* (%)	118 (100.0)	74 (100.0)	44 (100.0)	
FeNO (ppb)	34.2 ± 33.8 (22.0 [11.0–46.2])	37.4 ± 37.8 (25.0 [11.0–49.0])	28.8 ± 25.3 (19.5 [12.0–37.0])	0.568 (MW)
Positive skin prick test, *n* (%)	107.0 (90.7)	67.0 (90.5)	40.0 (90.9)	0.611 (FE)
GINA step, *n* (%)				
Step 1–2 (Mild)	51 (43.2)	38 (51.4)	13 (29.5)	
Step 3 (Moderate)	45 (38.2)	29 (39.2)	16 (36.4)	0.005 (FE) *
Step 4–5 (Severe)	22 (18.6)	7 (9.5)	15 (34.0)	
ACQ-6 score	0.61 ± 0.72 (0.50 [0.00–1.00])	0.49 ± 0.55 (0.28 [0.00–0.80])	0.82 ± 0.90 (0.65 [0.03–1.19])	0.032 (MW) *
Asthma control, *n* (%)				
Well controlled (ACQ < 0.75)	27 (22.9)	14 (18.9)	13 (29.5)	
Partially controlled	79 (66.9)	55 (74.3)	24 (54.5)	0.076 (FE)
Uncontrolled (ACQ ≥ 1.5)	12 (10.2)	5 (6.8)	7 (15.9)	
**Biochemical parameters**				
Serum HDL-c (mg/dL)	48.5 ± 9.72 (48.0 [40.7–55.0])	48.5 ± 9.82 (48.0 [40.7–55.0])	48.3 ± 9.66 (48.5 [40.2–55.5])	0.916 (t)
Serum total cholesterol (mg/dL)	149.1 ± 26.7 (145.0 [130.7–167.0])	150.2 ± 26.9 (145.0 [132.7–164.2])	147.3 ± 26.6 (144.0 [128.0–172.0])	0.925 (MW)
Serum triglycerides (mg/dL)	99.6 ± 61.2 (78.5 [60.7–117.2])	107.8 ± 71.1 (76.5 [61.7–126.7])	85.9 ± 35.8 (83.0 [58.2–106.5])	0.849 (MW)
Serum LDL-c (mg/dL)	80.7 ± 22.0 (77.3 [66.2–95.3])	80.0 ± 22.7 (77.3 [65.8–90.5])	81.7 ± 20.8 (81.0 [67.0–98.8])	0.431(MW)
Serum uric acid (mg/dL)	4.6 ± 1.17 (4.50 [3.87–5.40])	4.77 ± 1.30 (4.5 [3.95–5.52])	4.48 ± 0.91 (4.5 [3.82–5.00])	0.168 (t)
Serum 25-OH vitamin D (ng/mL)	19.4 ± 5.76 (19.1 [15.3–22.7])	20.7 ± 6.19 (20.2 [16.0–25.4])	17.4 ± 4.31 (16.8 [14.1–21.7])	0.001(t) *
Vitamin D status, *n* (%)				
Deficient	66 (55.9)	35 (47.3)	31 (70.5)	
Insufficient	44 (37.3)	31 (41.9)	13 (29.5)	0.012 (FE)
Sufficient	8 (6.8)	8 (10.8)	0 (0.0)	
**Pulmonary function**				
FEV1% predicted	99.1 ± 16.7 (96.3 [86.9–108.9])	98.7 ± 18.0 (96.3 [87.33–108.32])	99.8 ± 14.4 (96.9 [86.6–108.9])	0.849 (MW)
FVC% predicted	107.3 ± 14.8 (106.4 [100.0–113.5])	105.1 ± 15.3 (106.4 [99.5–111.8])	111.1 ± 13.4 (107.0 [101.7–119.7])	0.568 (MW)
FEV1/FVC% predicted	81.4 ± 8.38 (82.0 [75.0–89.2])	81.6 ± 9.10 (82.0 [75.7–90.0])	81.0 ± 7.09 (83.0 [74.2–86.0])	0.740 (MW)

Continuous variables are reported as mean ± SD (median [P25–P75]), and categorical variables are reported as n (%). Test abbreviations (in *p-*value column): t = independent sample *t*-test; MW = Mann–Whitney U test; χ^2^ = Chi-square test; FE = Fisher’s exact test. * *p* < 0.05. ACQ-6, Asthma Control Questionnaire 6-item; BMI: body mass index; FeNO: fractional exhaled nitric oxide; FEV1: forced expiratory volume in the first second; FVC: forced vital capacity; GINA: Global Initiative for Asthma; HDL-c: high-density lipoprotein cholesterol; LDL-c: low-density lipoprotein cholesterol; SD: standard deviation; P25–P75: 25th–75th percentile.

### 3.2. Combined-Sample Hierarchical Regression Models

Three parallel hierarchical regression models were developed using the same predictors. The results are summarized in Table 2.

**Table 2:** Hierarchical multiple linear regression models predicting pulmonary function in children and adolescents with asthma.

Variables	FEV1% Predicted *n* = 118			FVC% Predicted *n* = 118			FEV1/FVC% Predicted *n* = 117 ^a^		
	B (β)	BCa 95% CI	*p* Bootstrap BCa *	B (β)	BCa 95% CI	*p* Bootstrap BCa *	B (β)	BCa 95% CI	*p* Bootstrap BCa *
**Block 1:** **Demographic and anthropometric covariates**									
Sex (male = 1)	2.047 (0.059)	−4.045, 8.218	0.510	6.757 (0.221)	1.667, 11.683	0.015 *	−0.864 (−0.050)	−3.897, 2.139	0.572
BMI z-score	0.656 (0.301)	0.275, 1.040	0.001 *	0.567 (0.293)	0.231, 0.903	0.001 *	−0.971 (−0.148)	−2.185, 0.233	0.113
*R*^2^ (Adjusted *R*^2^)	0.091 (0.075)			0.123			0.023 (0.006)		
*p* of Block 1	0.004			<0.001			0.269		
**Block 2:** **Clinical, metabolic, and pubertal predictors**									
Sex (male = 1)	4.972 (0.144)	−0.671, 10.508	0.080	9.073 (0.296)	4.401, 13.925	<0.001 *	−0.150 (−0.009)	−3.329, 2.974	0.926
BMI z-score	0.566 (0.259)	0.222, 0.901	<0.001 *	0.494 (0.255)	0.175, 0.813	0.002 *	−0.676 (−0.103)	−1.888, 0.553	0.274
ACQ-6 score	−5.165 (−0.222)	−9.422, −1.249	0.011 *	−2.702 (−0.131)	−6.289, −0.412	0.069	−1.916 (−0.165)	−3.900, 0.873	0.081
Serum HDL-c (mg/dL)	0.247 (0.143)	−0.066, 0.527	0.087	0.110 (0.072)	−0.107, 0.331	0.346	0.064 (0.074)	−0.098, 0.221	0.438
Mid-puberty (Tanner III) ^b^	−11.098 (−0.304)	−17.480, −4.412	<0.001 *	−9.208 (−0.284)	−14.66, −3.992	0.002 *	−1.982 (−0.108)	−5.828, 1.790	0.309
Late puberty (Tanner IV)	−9.160 (−0.255)	−15.770, −2.371	0.005 *	−9.858 (−0.309)	−15.939, −3.910	0.004 *	−0.433 (−0.024)	−4.162, 3.352	0.819
Total *R*^2^ (Adjusted *R*^2^)	0.254 (0.214)			0.247 (0.206)			0.061 (0.010)		
Δ*R*^2^ (Block 2 vs. Block 1)	0.163			0.124			0.038		
Cohen’s *f*^2^	0.218			0.165			0.040		
*p* of Δ*R*^2^	<0.001			0.002			0.353		
*p* of full model	<0.001			<0.001			0.318		
SEE %pred	14.875			13.248			8.364		

Abbreviations: ACQ-6: Asthma Control Questionnaire 6-item; BCa: bias-corrected and accelerated; BMI: body mass index; CI: confidence interval; FEV1: forced expiratory volume in 1 s; FVC: forced vital capacity; HDL-c: high-density lipoprotein cholesterol. ^a^ FEV1/FVC model was performed after exclusion of one extreme outlier (Cook’s D = 6.875). ^b^ Reference category: pre-/early puberty (Tanner I–II, *n* = 46). Mid-puberty (Tanner III, *n* = 35) and late puberty (Tanner IV, *n* = 37). Bootstrap BCa estimates were based on 5000 resamples. * *p* < 0.05.

#### 3.2.1. FEV1% Predicted

The full FEV1% predicted model was statistically significant and explained 25.4% of the variance (adjusted R^2^ = 0.214, Cohen’s f^2^ = 0.218; Table 2). Mid-puberty (Tanner III) and late puberty were each associated with a clinically meaningful reduction on FEV1, corresponding to decreases of 11.1 and 9.2 percentage points, respectively, relative to the early-pubertal reference category. The ACQ-6 score was inversely associated with FEV1% predicted, whereas the BMI z-score was positively associated, and HDL-c showed a positive but non-significant tendency. The full coefficients, bootstrap BCa confidence intervals, and *p*-values are presented in Table 2.

#### 3.2.2. FVC% Predicted

The full FVC% predicted model was statistically significant and explained 24.7% of the variance (adjusted *R*^2^ = 0.206, Cohen’s *f*^2^ = 0.165). Male sex was the strongest predictor. Mid- and late puberty (*p* = 0.002) were both negatively associated with FVC% predicted, and the BMI z-score was positively associated. The ACQ-6 score showed a marginal statistical tendency. Full estimates are reported in Table 2.

#### 3.2.3. FEV1/FVC% Predicted

In contrast to the FEV1% and FVC% models, the combined FEV1/FVC% predicted model did not reach statistical significance (adjusted *R*^2^ = 0.010; n = 117 after exclusion of one influential observation; see Section 2.5.4 and Table 2). None of the predictors, including pubertal stage, showed independent associations with the FEV1/FVC ratio.

#### 3.2.4. Comparative Pattern: Volumetric vs. Obstructive Effects

A consistent pattern emerged in the three combined-sample models: pubertal stage was negatively associated with both FEV1% and FVC% predicted (standardized β range: −0.255 to −0.309) but showed no association with the FEV1/FVC ratio. Similarly, the BMI z-score positively predicted both volumetric parameters but did not affect the ratio.

### 3.3. Sex-Stratified Analyses

The sex-stratified hierarchical regression analysis results are summarized in Table 3 (FEV1%), Table 4 (FVC%), and Table 5 (FEV1/FVC%).

**Table 3:** Sex-stratified hierarchical regression models predicting FEV1% predicted: bootstrap BCa estimates.

Predictor	Males (*n* = 73) ^a^			Females (*n* = 44)		
	B (β)	BCa 95% CI	*p* Bootstrap BCa *	B (β)	BCa 95% CI	*p* Bootstrap BCa *
BMI z-score	−2.276 (−0.194)	−4.930, 0.239	0.079	6.505 (0.429)	2.137, 11.092	0.006
ACQ-6 score	−5.212 (−0.171)	−12.409, 0.539	0.110	−4.853 (−0.306)	−8.512, 1.032	0.020 *
Serum HDL-c (mg/dL)	−0.105 (−0.061)	−0.456, 0.215	0.519	0.690 (0.463)	0.272, 1.122	0.003 *
Mid-puberty (Tanner III) ^b^	−14.246 (−0.394)	−23.227, −5.625	0.002 *	−9.048 (−0.283)	−20.123, 0.858	0.127
Late puberty (Tanner IV)	−16.245 (−0.417)	−25.758, −6.858	0.001 *	−5.424 (−0.188)	−15.272, 4.468	0.306
Total *R*^2^ (Adjusted *R*^2^)	0.251 (0.195)			0.339 (0.252)		
Cohen’s *f*^2^	0.265			0.380		
*p* of full model	0.001 *			0.006 *		

Abbreviations: ACQ-6: Asthma Control Questionnaire 6-item; BCa: bias-corrected and accelerated; BMI: body mass index; CI: confidence interval; HDL-c: high-density lipoprotein cholesterol. ^a^ One case with Cook’s distance = 6.875 was excluded due to extreme influence; n was reduced from 74 to 73. ^b^ Reference category: Tanner I–II. Bootstrap BCa estimates were based on 5000 resamples. * *p* < 0.05.

**Table 4:** Sex-stratified hierarchical regression models predicting FVC% predicted: bootstrap BCa estimates.

Predictor	Males (*n* = 74)			Females (*n* = 44)		
	B (β)	BCa 95% CI	*p* Bootstrap BCa *	B (β)	BCa 95% CI	*p* Bootstrap BCa *
BMI z-score	0.455 (0.287) ^a^	−1.403, 0.966 ^b^	0.004 *^b^	6.080 (0.431)	2.072, 10.950	0.005 *
ACQ-6 score	−6.353 (−0.230)	−13.064, −0.858	0.043 *	−0.137 (−0.009)	−3.557, 5.914	0.926
Serum HDL-c (mg/dL)	−0.034 (−0.022)	−0.328, 0.214	0.831	0.475 (0.341)	0.050, 0.988	0.052
Mid-puberty (Tanner III) ^c^	−11.664 (−0.355)	−20.000, −3.895	0.005 *	−10.880 (−0.365)	−21.452, −2.521	0.031 *
Late puberty (Tanner IV)	−10.588 (−0.299)	−19.679, −2.865	0.021 *	−13.525 (−0.504)	−21.559, −5.076	0.006 *
Total *R*^2^ (Adjusted *R*^2^)	0.284 (0.232)			0.310 (0.220)		
Cohen’s *f*^2^	0.229			0.349		
*p* of full model	<0.001 *			0.012 *		

^a^ Sensitivity analysis with one influential case excluded (Cook’s D = 3.008) showed that the BMI z-score effect in males was artifactual (β = −0.023, *p* = 0.839, n = 73); see Supplementary Table S2. The effects of the pubertal stage remained stable. ^b^ Apparent CI-p discrepancy in the male BMI z-score row: the bootstrap BCa interval (−1.403, 0.966) crosses zero despite a bootstrap p-value of 0.004. This reflects the strong skewness (bootstrap bias = −0.242; bootstrap standard error [SE] = 0.771) of the resampling distribution, which was driven by a single influential observation (Cook’s D = 3.008). The corresponding ordinary least squares (OLS) estimate (B = 0.455; SE = 0.165; t = 2.766; *p* = 0.007; OLS 95% CI: 0.127–0.784) is consistent with a positive association and is reported in full in Supplementary Table S5. After exclusion of the influential observation in the sensitivity analysis (Supplementary Table S2), the effect attenuated to β = −0.023 (*p* = 0.839); therefore, our substantive interpretation concludes that the BMI z-score has no robust association with FVC% in males. ^c^ Reference category: pre-/early puberty (Tanner I–II; males = 33, females = 13). Mid-puberty corresponded to Tanner III (males = 23; females = 12) and late puberty corresponded to Tanner IV (males = 18; females = 19). No participant had reached Tanner stage V at the time of recruitment. Abbreviations: ACQ-6: Asthma Control Questionnaire 6-item; BCa: bias-corrected and accelerated bootstrap; BMI: body mass index; CI: confidence interval; FVC: forced vital capacity; HDL-c: high-density lipoprotein cholesterol. * *p* < 0.05.

**Table 5:** Sex-stratified hierarchical regression models predicting FEV1/FVC% predicted: bootstrap BCa estimates.

Predictor	Males (*n* = 73) ^a^			Females (*n* = 44)		
	B (β)	BCa 95% CI	*p* Bootstrap BCa *	B (β)	BCa 95% CI	*p* Bootstrap BCa *
BMI z-score	−1.736 (−0.274)	−3.217, −0.194	0.015 *	1.335 (0.179)	−0.629, 3.270	0.157
ACQ-6 score	0.767 (0.047)	−2.836, 4.204	0.679	−3.698 (−0.474)	−5.709, −0.071	0.006 *
Serum HDL-c (mg/dL)	−0.048 (−0.052)	−0.273, 0.180	0.687	0.247 (0.336)	0.024, 0.460	0.028 *
Mid-puberty (Tanner III) ^b^	−1.927 (−0.099)	−6.797, 2.832	0.439	−2.439 (−0.155)	−8.190, 2.939	0.412
Late puberty (Tanner IV)	−4.029 (−0.191)	−9.473, 1.604	0.149	3.192 (0.226)	−1.395, 7.652	0.152
Total *R*^2^ (Adjusted *R*^2^)	0.090 (0.022)			0.354 (0.268)		
Cohen’s *f*^2^	0.034			0.508		
*p* of full model	0.264			0.004 *		

Abbreviations: ACQ-6: Asthma Control Questionnaire 6-item; BCa: bias-corrected and accelerated; BMI: body mass index; CI: confidence interval; FEV1: forced expiratory volume in 1 s; FVC: forced vital capacity; HDL-c: high-density lipoprotein cholesterol. ^a^ One case with Cook’s distance = 6.875 was excluded because of extreme influence (n was reduced from 74 to 73 in the male model). ^b^ Reference category: pre-/early puberty (Tanner I–II). Bootstrap BCa estimates were based on 5000 resamples. * *p* < 0.05.

#### 3.3.1. FEV1% Predicted Stratified by Sex

In males (*n* = 73, after exclusion of one observation; see Section 2.5.4), pubertal stage emerged as the most prominent predictor of FEV1% predicted, and both mid-pubertal and late puberty were significantly associated with lower values. In females (*n* = 44), the most significant predictors were serum HDL-c, BMI z-score, and the ACQ-6 score, all of which retained significance, whereas pubertal stage was not an independent predictor of FEV1% in this group. The full coefficients, bootstrap BCa confidence intervals, and *p*-values are presented in Table 3.

#### 3.3.2. FVC% Predicted Stratified by Sex

In males (*n* = 74), pubertal stage (mid- and late puberty) and the ACQ-6 were significant predictors of the FVC% predicted. The BMI z-score showed an apparently positive coefficient in the primary model; however, this estimate was unstable, as its bootstrap BCa interval crossed zero, and a pre-specified sensitivity analysis excluding a single influential observation (Cook’s D = 3.008) attenuated the effect to non-significance (Supplementary Tables S2 and S5). Therefore, we conclude that there is no robust association between the BMI z-score and FVC% in males and that the male FVC% pattern is anchored in the pubertal stage and the ACQ-6 score. In females (*n* = 44), pubertal stage was a significant predictor with a progressively larger effect across stages, and the BMI z-score was positively associated with FVC%, but the ACQ-6 score was not. Full estimates and details of the influential observation analysis are presented in Table 4 and its footnotes.

#### 3.3.3. FEV1/FVC% Predicted Stratified by Sex

In males (*n* = 73, after exclusion of one influential observation), the model did not reach overall statistical significance, and the BMI z-score was the only significant predictor. In females (n = 44), the model was statistically significant and showed a large effect size (Cohen’s *f*^2^ = 0.508); the ACQ-6 score (*β* = −0.474) and HDL-c (*β* = 0.336) were the strongest associated variables, although the HDL-c finding was only marginal after FDR correction (*q* = 0.065; Section 3.3.5). Full estimates are presented in Table 5; notably, the Tanner IV coefficients ran in opposite directions between the sexes (males: *β* = −0.191; females: *β* = 0.226), although this between-sex contrast should be interpreted as exploratory given that the formal sex x predictor interaction test did not reach statistical significance (Section 3.3.4).

#### 3.3.4. Formal Test of Sex-by-Predictor Interactions

In the full sample (*n* = 117), a hierarchical model with three interaction terms (sex × mid-puberty, sex × late puberty, sex × HDL-c) was used to formally evaluate whether the sex-specific patterns identified in the stratified analyses indicated any significant effect modification. The interaction block did not significantly improve the model fit (Δ*R*^2^ = 0.037; *p* = 0.178), and none of the individual interaction coefficients reached statistical significance under the bootstrap BCa estimation. The complete results of the interaction model are presented in Supplementary Table S3.

The interaction model also showed marked numerical instability: 1701 of the 5000 bootstrap replicates (34.0%) failed to converge, owing to the near-collinear configurations produced by the three product terms in some resamples. This high non-convergence rate is itself an indicator of limited statistical power for detecting interactions, given the number of estimated parameters relative to the sample size (10 parameters; 11.7 observations per parameter), and reinforces the hypothesis-generating interpretation of the sex-stratified findings (Section 4.6).

#### 3.3.5. Multiple-Testing Correction

Regarding the application of the pre-specified Benjamini–Hochberg false discovery rate correction (Section 2.5.3) to the 30 sex-stratified bootstrap BCa contrasts (Supplementary Table S6), of the 15 contrasts with raw *p* < 0.05, 10 retained significance under stringent FDR control (*q* < 0.05): the mid- and late-pubertal effects on FEV1% in males; the mid-pubertal effect on FVC% in males and the late-pubertal effect on FVC% in females; the BMI z-score and HDL-c in female FEV1%; the BMI z-score in male FVC%; the BMI z-score in female FVC%; the BMI z-score in male FEV1/FVC%; and the ACQ-6 in female FEV1/FVC%. A further six contrasts fell into the marginal zone (0.05 ≤ *q* < 0.10): the ACQ-6 in female FEV1% (*q* = 0.052), the ACQ-6 in male FVC% (*q* = 0.086), late-pubertal FVC% in males (*q* = 0.052), mid-pubertal FVC% in females (*q* = 0.066), HDL-c in female FVC% (*q* = 0.097), and HDL-c in female FEV1/FVC% (*q* = 0.065). Thus, the effects of pubertal stage on volumetric pulmonary function were robust in the combined sample and in several sex-stratified contrasts after FDR correction.

## 4. Discussion

Our study highlights that relevant associations were estimated from cross-sectional data and that no causal inferences regarding the effects of pubertal maturation, body composition, or HDL-c on pulmonary function are warranted from the present design. Therefore, the mechanistic interpretations developed below should be read as biologically plausible hypotheses consistent with the observed associations, not as demonstrated causal relationships. The terms “structural maturational” and “metabolic inflammatory” used throughout this section refer to candidate frameworks for organizing the observed sex-stratified associations rather than verified mechanisms operating in our study.

### 4.1. Pubertal Associations with Volumetric Lung Function in the Combined Sample

In the combined-sample analysis, pubertal stage showed a negative association with both FEV1% and FVC% predicted with similar magnitude (β range: −0.255 to −0.309) but did not influence the FEV1/FVC% ratio. This dissociation constitutes the principal physiological insight at the integrated level and corroborates the dysanaptic growth hypothesis during puberty [15,17]. If the effects of puberty were mostly associated with airway-specific obstructive mechanisms, FEV1% would be expected to show a larger negative association than FVC%, with a corresponding reduction in the FEV1/FVC ratio. Given the cross-sectional nature of this study, these findings should be interpreted as associations rather than as evidence of developmental effects. A recent multi-ethnic population-based study reported that more advanced pubertal stages were associated with higher FEV1 and FVC in both sexes among children from the general population [71]. The opposite direction observed in our asthmatic cohort most likely reflects methodological and population differences: that study expressed lung function as internally derived z-scores and adjusted for height gain in a predominantly non-asthmatic sample, whereas we analyzed percent-predicted value references to external equations in children with asthma. This contrast suggests that the association between pubertal stage and pulmonary function may differ between healthy children and those with asthma and underscores the need for caution when comparing results across reference frameworks.

### 4.2. Sex-Stratified Patterns of Association

Sex-stratified analyses suggested that the apparently uniform pubertal effect observed in the combined sample may reflect divergent patterns by sex. In males, pubertal stage was associated with lower FEV1% and FVC%, with associations that were somewhat more pronounced for FEV1% than for FVC%, possibly indicating a modest obstructive component in addition to the volumetric pubertal effect; however, the individual pubertal coefficients in the male FEV1/FVC% model did not reach statistical significance. In females, the pubertal stage was a significant predictor of FVC% predicted, with an increasing magnitude across stages, whereas the associations with FEV1% predicted were non-significant, resulting in a non-significant positive tendency in the FEV1/FVC ratio at advanced pubertal stages, in contrast to the negative tendency observed in males. These apparent between-sex differences should be interpreted with caution, as the formal sex × Tanner interaction test did not reach statistical significance in this study.

### 4.3. HDL Cholesterol as a Sex-Specific Correlate of Pulmonary Function in Females

The main finding of our study was the persistent positive association between serum HDL-c levels and pulmonary function, exclusively in females. This sex-specific HDL-c effect is consistent with multiple studies supporting estrogen-dependent modulation of HDL-c composition and function. A candidate mechanism for the female-specific HDL–pulmonary function association in our peripubertal cohort is estrogen-dependent modulation of HDL biogenesis: estradiol has been shown in adult populations to stimulate hepatic apolipoprotein A-I synthesis and increase the production of larger, ApoA-I-enriched HDL particles with enhanced cholesterol efflux capacity [72]. We emphasize that serum HDL-c concentration alone, as measured in our study, captures neither HDL particle size distribution nor HDL functionality (cholesterol efflux capacity, paraoxonase-1 activity, or other functional anti-inflammatory properties) and that direct evidence of estrogen-mediated effects on HDL biology in peripubertal pediatric asthma populations is currently lacking.

In addition to their direct effects on HDL biosynthesis, HDL particles possess significant anti-inflammatory and antioxidative properties, such as the modulation of Toll-like receptor signaling and endothelial function, potentially affecting the airway epithelium [36]. The relevance of these pathways in the pediatric population is supported by the established occurrence of dyslipidemia and other metabolic abnormalities in children with asthma, despite body mass index [39]. Consistent with the beneficial respiratory role of HDL-c in the pediatric range, a prospective study in the COPSAC_2000_ cohort reported that higher HDL-c levels were associated with lower specific airway resistance and reduced bronchial responsiveness in 7-year-old children; however, that study assessed prepubertal children, adjusted for sex rather than stratifying by it, and did not detect an association with spirometric volumes [73]. Our findings extend this evidence to the peripubertal period and suggest that the association between HDL-c and spirometric pulmonary function in pediatric asthma may be sex-specific. The extensive sexual dimorphism of lipid metabolism cannot be exclusively attributed to sex hormone exposure, as accumulating evidence suggests that sex differences in lipid homeostasis endure even when hormonal effects are considered and can be influenced by adiposity [40,41]. This complexity may explain why the HDL–pulmonary function association observed in our female group manifested specifically during pubertal maturation. Conversely, testosterone in males has been reported to be inversely associated with HDL-c levels [63] and may modify HDL-c functional properties, a finding consistent with the lack of HDL–pulmonary function associations in this group. The substantial impact of the HDL effect on FEV1/FVC% predicted in females *(β* = 0.336; *p* = 0.028), along with its considerable effect size in the corresponding stratified model (Cohen’s *f*^2^ = 0.508), suggests that HDL-c is preferentially associated with the obstructive pattern.

### 4.4. Adiposity-Related Dysanapsis Specific to Males

In contrast to the HDL-c pattern, the BMI z-score showed a male-specific negative association with FEV1/FVC%, consistent with the dysanaptic effects of adiposity on pediatric airway development [15,64]. This finding is consistent with male body composition during puberty, characterized by greater central adiposity and visceral fat accumulation, being associated with a more pronounced thoracic airway mismatch than the peripheral adiposity distribution observed in females [34]. Notably, in the male FVC% predicted model, the BMI z-score exhibited an apparently positive association in the primary analysis; however, sensitivity analyses indicated that this effect was predominantly influenced by a single case (Supplementary Table S2), reinforcing the interpretation that, in our male subgroup, adiposity is associated with the obstructive pattern (FEV1/FVC) rather than the total lung volume. In females, the BMI z-score was positively associated with FEV1% and FVC%, without association with the FEV1/FVC% ratio, a pattern consistent with sex-differential body composition–pulmonary function relationships.

### 4.5. Asthma Symptom Control as a Sex-Specific Predictor of Airflow Obstruction

The significant association of the ACQ-6 score with FEV1/FVC% only in the female group suggests a sex-differential pattern between asthma symptom control and airflow obstruction, rather than a uniform effect across sexes. This finding is consistent with prior reports from the NIH/NHLBI Severe Asthma Research Program (SARP), in which adolescent females in late pubertal stages exhibited lower post-bronchodilator FEV1% and FVC%, as well as worse ACQ-6 scores than males of a similar age [9]. In our study, the substantial proportion of female participants in Tanner stage IV (43.2%) suggests that, in this subgroup, circulating estrogen levels may already approximate adult ranges, providing biological plausibility for the emergence of estrogen-mediated mechanisms in the pediatric population.

Although direct measurements of sex steroids were not performed in our study, multiple non-mutually exclusive pathways may explain the female-specific predictors of pulmonary function identified in our regression models. First, estrogen enhances type 2-skewed immune responses, with multiple lines of evidence demonstrating increased IL-4 and IL-13 production, IgE class switching in B cells, and eosinophil recruitment to the lungs in response to physiological levels of estrogen [74]. Consistent with this immunological aspect, peripheral blood lymphocyte profiles in postpubertal females transition to a type 2-dominant pattern during the luteal phase of the menstrual cycle, characterized by selectively elevated IL-4 production without parallel changes in IFN-γ, along with an expansion of cytotoxic T-cell and natural killer cell populations [75]. Second, estrogen non-genomically stimulates mast cells through membrane-bound estrogen receptor-α, instigating calcium-dependent degranulation and enhancing the IgE-mediated release of allergic mediators [76]. Third, estrogen modulates airway smooth muscle responsiveness in a context-dependent manner, with experimental evidence indicating both acute bronchodilation via cAMP/PKA-mediated reduction in intracellular calcium [22] and increased hyperresponsiveness in chronically inflamed airways with elevated estrogen receptor expression [77]. Although these mechanisms have been predominantly elucidated in adult populations and the menstrual cycle phase was not evaluated in our peripubertal patients, the convergence of these pathways during pubertal maturation provides a biologically plausible framework for the differential associations observed in our female-stratified models.

### 4.6. An Exploratory Interpretation of Sex-Differential Patterns

Integrating our exploratory findings with the existing literature, the sex-stratified patterns may be tentatively interpreted as reflecting different patterns of association by sex, although we emphasize that the formal interaction test did not support sex-specific effect modification and that this interpretation is hypothesis-generating rather than being established. In males, more advanced pubertal stages were associated with lower FEV1% values, a pattern compatible with dysanaptic growth, and the BMI z-score was associated with airflow limitation. In females, the stratified analyses suggested a different pattern, in which HDL-c was positively associated with FEV1% and FEV1/FVC%, and asthma symptom control (ACQ-6) was inversely associated with FEV1/FVC%. These associations are biologically plausible, considering the documented effects of estrogen on type 2 inflammation [74], mast cell reactivity [76], and airway smooth muscle responsiveness [77], as discussed in Section 4.5. However, because sex hormones, inflammatory markers, and HDL functionality were not measured, these interpretations remain speculative and require confirmation in adequately powered studies with direct mechanistic assessments.

### 4.7. Clinical and Research Implications

Beyond the mechanistic interpretation outlined above, our findings have several practical implications. Sex-stratified studies require systematic attention in pediatric asthma research, particularly when investigating the metabolic, inflammatory, or maturational factors of lung function. From a clinical perspective, our results suggest that the variables most relevant for understanding pulmonary function in pediatric males and females may differ; however, the efficacy of targeting specific parameters to enhance respiratory outcomes requires validation through interventional studies with larger sample sizes.

From a nutritional and metabolic perspective, HDL-c, BMI z-score, and vitamin D status are biomarkers responsive to dietary and lifestyle factors, situating our findings within the broader context of nutrition and pediatric respiratory health. However, the present study did not assess dietary intake, and the following considerations are therefore contextual rather than inferences drawn from our data. The female-specific positive association of HDL-c with pulmonary function may motivate future studies to examine whether nutritional exposures that modify HDL-c are relevant to respiratory outcomes. For context, Mediterranean diet-based interventions in children have been associated with modest increases in HDL-c [78], and adherence to the Mediterranean dietary pattern has been associated with a protective role against asthma symptoms [79,80] and reduced airway inflammation [81]. Whether such dietary exposures have sex-differential effects on pulmonary function in pediatric asthma is unknown and would require prospective diet-assessed studies to address.

In contrast, the marked sex-related disparity in vitamin D status observed in our study, in which no female participant achieved sufficiency, situates our finding within an actively debated area of pediatric asthma research. Lower 25-hydroxyvitamin D concentrations have been consistently associated with greater asthma severity and worse symptom control in pediatric populations [82]. Similarly, interventional studies with vitamin D supplementation have reported a diminished risk of severe exacerbations [83], although the effects on spirometry results have been inconsistent across trials [84]. These associations between vitamin D and pulmonary function may be influenced by multiple factors, including body composition. In children with mild asthma, a positive association between serum 25-hydroxyvitamin D and FVC% and FEV1% has been reported only in normal-weight participants, with no comparable effect in those who were overweight or with obesity [85]. Moreover, vitamin D deficiency is independently amplified by both pubertal maturation and adiposity, particularly in females in early Tanner stages [86]. In summary, these observations suggest that the relevance of vitamin D for pulmonary function in pediatric asthma may become most apparent when evaluated in conjunction with metabolic, inflammatory, and pubertal factors, precisely the type of integrated, sex-stratified assessment that our exploratory finding suggest may be valuable in future research. Subsequent research should evaluate vitamin D levels, dietary intake, and supplementation as potential moderators of pulmonary function trajectories during puberty, including their potential sex-related differences.

### 4.8. Limitations

This study has several limitations. First, the cross-sectional design precludes causal inference regarding the relationship between pubertal maturation and lung function trajectories; longitudinal studies with repeated spirometry during pubertal transition are necessary to determine the temporal patterns. Second, we did not quantify circulating sex hormones (testosterone, estradiol, and dehydroepiandrosterone sulfate), nor did we evaluate the menstrual cycle phase in postmenarchal females. Direct quantification of these hormonal substrates would enable a formal assessment of the suggested estrogen-mediated mechanisms.

Third, although our sample size was sufficient for the primary analyses, it limited the statistical power to formally validate sex-by-predictor interactions in regression analyses. The formal interaction test was not significant, consistent with the established challenge of identifying interaction effects in observational designs, which generally require considerably larger sample sizes than those required for main effects [66]. A particularly important limitation is the numerical instability of the interaction model: 34.0% of the 5000 bootstrap replicates failed to converge because of the near-collinear configurations introduced by the three product terms. This high non-convergence rate is not merely a technical detail but a substantive constraint, as it indicates that the interaction analysis is underpowered and that its confidence intervals are unstable. Accordingly, the formal interaction test should be regarded as inconclusive rather than as evidence against effect modification, and the sex-stratified findings must be interpreted strictly as exploratory, hypothesis-generating evidence that requires confirmation in adequately powered prospective cohort studies.

Fourth, the study sample was unbalanced by sex (74 males and 44 females), reflecting the well-documented male predominance of asthma in the prepubertal and peripubertal age groups. Consequently, the female subsample (*n* = 44) was at the lower limit of Harrell’s suggested observations-per-parameter ratio [69], which reduced the precision of female-specific estimates. Several female-stratified coefficients were accompanied by relatively wide bootstrap BCa confidence intervals, and the limited number of observations per estimated parameter raised the possibility of model overfitting in this subgroup. Therefore, these estimates should be regarded as the least stable and interpreted with caution. Because the cohort was recruited consecutively and is now complete, this imbalance could not be corrected, and confirmation in a larger and more sex-balanced sample is warranted. Additionally, participants were aged 6 to 17 years, and no participant had reached Tanner stage V at the time of recruitment; moreover, the reference category was composed predominantly of Tanner II participants. Consequently, the pubertal contrast estimated in this study reflects differences between the early and mid-/late-pubertal stages rather than the complete pubertal transition, and the findings cannot be extrapolated to fully mature adolescents. Studies enrolling a larger proportion of older adolescents who have completed pubertal development are needed to characterize the correlates of pulmonary function in the later stages of puberty. Fifth, two separate observations exhibited Cook’s distance greater than one in the different regression models. However, both exclusions were based on objective influence diagnostics, analyzed using sensitivity analyses, and properly disclosed.

Sixth, percent-predicted spirometry values were calculated using the NHANES III reference equations [60], which were natively supported by the spirometry equipment used. Although NHANES III is extensively utilized in clinical and research settings and includes Mexican-American sex-specific coefficients, longitudinal data from healthy Mexican children residing in Mexico [87] have shown systematically higher FEV1 and FVC than predicted by the NHANES III Mexican-American equations (mean z-scores of approximately +0.23 for FEV1 and +0.21 for FVC, equivalent to approximately 70 mL above the predicted value for both volumes). This suggests that residual ethnic and environmental variance specific to Mexican children residing in Mexico is not fully absorbed by the Mexican-American adjustment in NHANES III and that the percent-predicted values reported in our study may be modestly attenuated relative to a Mexico-specific reference. Furthermore, our cohort was recruited in Mexico City at approximately 2250 m above sea level, where baseline spirometry values may differ from sea-level cohorts owing to atmospheric pressure and air density effects on flow-dependent measures, although the same study found that altitude adjustment of air density did not reduce the observed differences in PEF between Mexican children and the NHANES III Mexican-American reference. The more recent Global Lung Function Initiative (GLI) 2012 equations [88] have been established as the international standard and offer multi-ethnic reference values across a broader age range (3–95 years old). However, the GLI 2023 race-neutral equations have been shown to underestimate predicted lung volumes in children and adolescents relative to GLI 2012 [89], complicating the choice between current alternatives. Whether the magnitude (rather than the direction) of the sex-specific effects identified in our study is sensitive to reference equation choice could not be evaluated within the scope of this analysis; future analyses with raw spirometric values and parallel application of NHANES III, GLI 2012, and Mexico-specific reference equations are warranted.

Seventh, while serum 25-hydroxyvitamin D was evaluated at a single time point and showed marked sex-related differences, seasonal variation, sun exposure, and dietary vitamin D intake were not examined. Nonetheless, the identified sex differences in vitamin D levels suggest that future analyses incorporating vitamin D as a potential effect modifier are necessary.

Eighth, the Spanish-language ACQ-6 used in this study [58] was originally validated in adult populations, and no formal pediatric Spanish-language validation of the ACQ-5/ACQ-6 exists, despite the original English version of the ACQ being validated in pediatric populations [90]. However, the original English ACQ has demonstrated robust internal consistency (Cronbach’s α typically reported as 0.85–0.93 across populations [56]), and the validated Spanish adult version showed equivalent psychometric properties [58]. Nevertheless, in the absence of a formal pediatric Spanish-language validation, the associations identified using the ACQ-6 in this research should be considered tentative and interpreted with caution. Importantly, because the ACQ-6 was one of the strongest predictors in the female-stratified models (notably for FEV1/FVC%), any measurement imprecision in this instrument could directly affect the corresponding coefficient estimates; therefore, the ACQ-6-based associations in the smaller female subgroup should be regarded as particularly provisional.

Ninth, our study did not include a healthy non-asthmatic control group, as our primary focus was on elucidating sex-related patterns in pediatric asthma pathogenesis. Thus, we were unable to determine whether the observed sex-related patterns were exclusive to asthma or indicative of general developmental physiology of pulmonary function during puberty. Subsequent research involving healthy individuals would help distinguish disease-specific from physiological dimorphisms.

Tenth, our analytic plan involved 30 sex-stratified hypothesis tests (5 predictors × 3 outcomes × 2 sexes), generating a substantial family-wise error risk. Although the Benjamini–Hochberg FDR correction (Supplementary Table S6) preserved the dominant pubertal-stage findings and several of the BMI z-score and HDL-c associations, one of the central female-specific findings, HDL-c as a correlate of FEV1/FVC%, fell into the marginal zone (*q* = 0.065) and did not survive stringent FDR control at *q* < 0.05. This further indicates that the female HDL-c–FEV1/FVC% association, in particular, requires confirmation before any sex-differential interpretation can be considered to be established.

A further limitation pertains to the nutritional assessment. Although HDL-c and the BMI z-score are metabolically significant and directly related to the nutritional physiology of puberty, the study did not account for dietary intake, dietary patterns, physical activity, or direct measures of HDL functionality and lipid quality. Consequently, the nutritional conclusions drawn here are indirect, and the dietary considerations discussed in Section 4.7 (such as the Mediterranean dietary pattern and vitamin D status) should be viewed as hypotheses connecting the observed metabolic associations to modifiable nutritional exposures, rather than findings supported by primary dietary data. Future research incorporating validated dietary questionnaires, objective activity measurements, and HDL functional assays is necessary to substantiate the nutritional aspect of these associations. Finally, the study was conducted in a single major pediatric hospital in Mexico City, which may restrict its applicability to other Latin American populations, rural environments, varying socioeconomic levels, or individuals residing at sea level; therefore, the residual effects of altitude on baseline spirometry values cannot be disregarded.

## 5. Conclusions

This study of Mexican children and adolescents with asthma identified apparently divergent patterns in the correlates of pulmonary function. In males, pubertal stage and BMI z-score were the dominant associated variables; in females, serum HDL-c, BMI z-score, and asthma symptom control were the principal associated variables. Pubertal-stage effects in volumetric lung function were robust in the combined sample and in at least one volumetric outcome in each sex. However, the formal sex-by-predictor interaction test did not reach statistical significance, indicating that these sex-related patterns require confirmation in larger adequately powered cohorts; nevertheless, the consistency and biological plausibility of the stratified findings provide an exploratory basis that can guide future research. These results demonstrate the value of sex-stratified analysis in pediatric asthma and identify HDL-c, body composition, and symptom control as sex-specific candidate correlates of pulmonary function whose potential sex-related differences warrant prospective and interventional investigations, including direct measures of sex hormones, HDL functionality, and integrated nutritional assessment.

## Data Availability

The de-identified datasets generated and analyzed during the current study are available from the corresponding author upon reasonable request, subject to compliance with the institutional research data-sharing policies of the Hospital Infantil de México Federico Gómez and additional ethical review when required, given the pediatric nature of the cohort.

## Supplementary Materials

The following supporting information can be downloaded at: https://www.mdpi.com/article/10.3390/nu18121885/s1; Table S1: Exploratory analyses with additional metabolic predictors: summary of findings; Table S2: Sensitivity analysis: male FVC% predicted model with and without one influential observation (Cook’s D = 3.008) bootstrap BCa estimates; Table S3: Hierarchical regression model with sex × predictor interaction terms in the full sample (*n* = 117): bootstrap BCa estimates; Table S4: Multicollinearity diagnostics for the three primary hierarchical regression models in the combined sample (Block 2); Table S5: Sex-stratified hierarchical regression models predicting FVC% Predicted: comparison of OLS and bootstrap BCa estimates; Table S6: Multiple-testing correction (Benjamini–Hochberg FDR) for sex-stratified bootstrap BCa *p*-values across the three pulmonary function outcomes.

## Abbreviations

The following abbreviations are used in this manuscript:

ACQ-6	Asthma Control Questionnaire, 6-item version
BCa	Bias-corrected and accelerated
BMI	Body mass index
CI	Confidence interval
FEV1	Forced expiratory volume in 1 s
FVC	Forced vital capacity
GINA	Global Initiative for Asthma
FeNO	Fractional exhaled nitric oxide
HDL-c	High-density lipoprotein cholesterol
NHANES III	Third National Health and Nutrition Examination Survey
SD	Standard deviation
25(OH)D	25-hydroxyvitamin D
