# Supplementary material for: Sex-Stratified Correlates of Pulmonary Function in Mexican Children and Adolescents (6–17 Years) with Asthma: An Exploratory Analysis of HDL Cholesterol, BMI, and Pubertal Stage
Source: Nutrients. 2026 Jun 11;18(12):1885. doi: 10.3390/nu18121885 (PMC13305537; doi:10.3390/nu18121885)
Supplement: Supplementary file 1 [file nutrients-18-01885-s001.zip › nutrients-4335163-supplementary.pdf]

Supplementary Materials

Title: Sex-Stratified Correlates of Pulmonary Function in Mexican Children and Adolescents (6–17 Years) with Asthma: An Exploratory Analysis of HDL Cholesterol, BMI, and Pubertal Stage

Authors: Nayely Reyes-Noriega[1], José J. Leija-Martínez[2], Fausto Sánchez-Muñoz[3], Adrián Hernández-Díaz Couder[4], Claudia Tavera Alonso[5], Santiago Villafaña[6], Darío Jorge Mario Molina Díaz[7], Blanca E. Del-Río-Navarro[1]*, Fengyang Huang[8]*

Table S1. Exploratory Analyses with Additional Metabolic Predictors: Summary of Findings.

In addition to the primary predictors retained in the main analyses, several metabolic biomarkers were initially explored to characterize the broader metabolic landscape of pulmonary function.

Predictor Explored	Outcome Tested	Finding	Status in Final Manuscript
Serum 25-OH Vitamin D	FVC% predicted	Showed marginal positive association in Block 2 (β = 0.175; p = 0.051) that was attenuated to non-significance after the addition of pubertal stage variables (β = 0.099; p = 0.268), suggesting confounding by pubertal stage.	Excluded from the main model; mentioned in the Discussion.
Serum total cholesterol	FVC% predicted	Showed a positive trend in Block 2 (β = 0.156; p = 0.072) that remained marginal after pubertal adjustment (β = 0.140; p = 0.094).	Excluded from the main model.
Serum uric acid	FEV1 (liters, absolute)	Initially showed apparent positive association (B = 0.158; p = 0.003).	Methodological exploration.
Serum uric acid	FEV1% predicted	The effect was attenuated to non-significance (B = 0.765; p = 0.595; BCa 95% CI: -2.082 to 3.611) when the outcome was expressed as percent-	Excluded; planned for a separate methodological manuscript on growth-adjustment in pediatric metabolic-respiratory research.

		predicted with adjustment for pubertal stage.	
Triglyceride/HDL ratio	FEV1% predicted	Did not show independent association after adjusting for HDL cholesterol alone.	Excluded from the main model.
GINA severity step	FEV1%, FVC%, FEV1/FVC% predicted	Did not show independent association after adjusting for the ACQ-6 score (which captures more granular control information).	Excluded from the main model.

These exploratory findings collectively support the focused predictor specification used in the main analyses (sex, BMI z-score, ACQ-6, HDL-c, and pubertal stage), as additional metabolic predictors did not contribute independent variance after appropriate adjustment for somatic growth and pubertal maturation.

Table S2: Sensitivity Analysis: Male FVC% Predicted Model with and without One Influential Observation (Cook's D = 3.008). Bootstrap BCa Estimates

Predictor	Primary Model (n= 74) [a]			Sensitivity Analysis (n=73) [a]		
	**B (β)**	**BCa 95% CI**	***p* boot**	**B (β)**	**BCa 95% CI**	***p* boot**
BMI z-score	0.455 (0.287)	-1.403, 0.966	0.004*	-0.227 (-0.023)	-2.554, 2.204	0.839
ACQ-6 score	-6.353 (-0.230)	-13.064,-0.858	0.043*	-6.021 (-0.233)	-12.504, -1.029	0.052
Serum HDL-c (mg/dL)	-0.034 (-0.022)	-0.328, 0.214	0.831	-0.064 (-0.044)	-0.361, 0.218	0.684
Mid-puberty (Tanner III)	-11.664 (-0.355)	-20.000, -3.895	0.005*	-11.375 (-0.370)	-19.782, -3.801	0.007*
Late puberty (Tanner IV)	-10.588 (-0.299)	-19.679, -2.865	0.021*	-10.923 (-0.330)	-20.252, -2.762	0.018*
Total R^2 (Adjusted R^2)	0.284 (0.232)			0.180 (0.119)		
Cohen's f^2	0.229			0.211		
p of full model	<0.001*			0.018*		

[a] One case with Cook's distance = 3.008 (mild to extreme influence) was excluded from the sensitivity analysis. Notably, the BMI z-score effect was attenuated from a positive significant association (β = 0.287; $p = 0.004$) to near-zero (β = -0.023; $p = 0.839$) upon exclusion of the influential case, indicating that this association in the primary model was largely driven by a single observation only. The effects of pubertal stage remained robust across both analyses. *$p < 0.05$

Table S3. Hierarchical Regression Model with Sex × Predictor Interaction Terms in the Full Sample (n = 117): Bootstrap BCa Estimates.

Predictor	B	β	BCa 95% CI	*p*
Block 1: Demographic covariates				
Sex (male=1)	3.665	0.112	-2.017, 9.511	0.211
BMI z-score	0.024	0.012	-1.740, 2.992	0.882
ACQ-6 score	-5.496	-0.249	-10.862, -0.593	0.026*
Serum HDL-c (mg/dL)	0.305	0.186	0.052, 0.543	0.020*
R^2 Block 1 = 0.082; F(4,112) = 2.517; p = 0.045				
Block 2: Pubertal stage added				
Sex (male=1)	4.837	0.147	-0.942, 10.524	0.103
BMI z-score	0.041	0.020	-1.507, 1.841	0.795
ACQ-6 score	-5.305	-0.240	-10.129, -1.106	0.022
Serum HDL-c (mg/dL)	0.222	0.136	-0.040, 0.462	0.096
Mid-puberty (Tanner III)	-10.845	-0.312	-17.673, -4.211	0.003*
Late puberty (Tanner IV)	-9.314	-0.272	-16.222, -2.219	0.018*
R^2 Block 2 = 0.174; ΔR^2 = 0.092; ΔF(2,110) = 6.129; p = 0.003				
Block 3: Sex x Predictor interaction terms added				
Sex (male=1)	-1.610	-0.049	-14.641, 12.731	0.803
BMI z-score	0.020	0.009	-1.506, 2.135	0.908
ACQ-6 score	-5.901	-0.267	-10.781, -1.414	0.013*
Serum HDL-c (mg/dL)	-0.008	-0.005	-0.359, 0.317	0.958
Mid-puberty (Tanner III)	-15.087	-0.434	-24.007, -5.721	0.001*
Late puberty (Tanner IV)	-14.964	-0.437	-24.309, -5.272	0.002*
Sex x Mid-puberty	8.768	0.167	-5.904, 22.808	0.260
Sex x Late puberty	11.931	0.277	-2.685, 25.261	0.125
Sex x Serum HDL-c	0.469	0.173	-0.134, 1.075	0.095
R^2 Block 3 = 0.211; ΔR^2 = 0.037; ΔF(3,107) = 1.667; p = 0.178				
Adjusted R^2 Block 3 = 0.145; SEE = 14.78; Cohen's f^2 (interactions) = 0.047				

Continuous predictors (BMI z-score, ACQ-6, and HDL-c) were mean-centered prior to creation of interaction terms to minimize collinearity. The sex variable was retained as uncentered (binary). The interaction block did not significantly improve the model fit ($\Delta R^2 = 0.037$; $p = 0.178$). Only 3,299 of 5,000 bootstrap replicates achieved convergence in the full interaction model, indicating numerical instability attributable to limited statistical power for detecting small-magnitude interactions (Cohen's $f^2 = 0.047$, requiring ~250 participants for adequate power). Note: The block structure of this interaction model differs from that of the primary stratified analyses (Tables 3-5): Block 1 here includes sex, BMI z-score, ACQ-6, and HDL-c (the four primary main-effect predictors retained for the interaction test), while Block 2 adds the pubertal stage variables and Block 3 adds the three interaction terms (sex × mid-puberty, sex × late-puberty, sex × HDL-c). This structure was chosen so that the formal test of effect modification (Block 3 ΔR^2) isolates the contribution of the interaction terms beyond the main effects of all key predictors, in keeping with established moderation analysis conventions [51].

Table S4. Multicollinearity Diagnostics for the Three Primary Hierarchical Regression Models in the Combined Sample (Block 2)

Predictor	FEV1 % VIF	FVC % VIF	FEV1/FVC % VIF	Tolerance (range)
Sex (male=1)	1.099	1.099	1.107	0.901-0.910
BMI z-score	1.020	1.020	1.153	0.867-0.980
ACQ-6 score	1.075	1.075	1.078	0.928-0.949
Serum HDL-c (mg/dL)	1.057	1.057	1.164	0.859-0.946
Mid-puberty (Tanner III)	1.266	1.266	1.283	0.779-0.790
Late puberty (Tanner IV)	1.348	1.348	1.347	0.742-0.756

All variance inflation factors (VIF) were below 1.4, well within the conventional threshold of acceptable multicollinearity (VIF < 5). The tolerance values exceeded 0.74 across all models, which was well above the 0.20 threshold. The maximum condition index across the three models reached 14.6, which is below the 30 threshold for problematic collinearity.

Table S5. Sex-Stratified Hierarchical Regression Models Predicting FVC%: Comparison of Ordinary Least Squares (OLS) and Bootstrap BCa Estimates.

This supplementary table is provided to allow direct inspection of the OLS reference estimates alongside the bootstrap BCa results presented in Table 4. The OLS estimates assume normality of residuals and are reported only as a methodological reference; primary inference relies on the bootstrap BCa intervals because residuals deviated from normality in the majority of models (Section 2.5.3).

Males (n= 74):

Predictor	B	SE	β	t	OLS 95% CI	*p* (OLS)	BCa 95% CI	*p* (BCa)
BMI z-score	0.455	0.165	0.287	2.766	0.127, 0.784	0.007*	-1.403, 0.966	0.004
ACQ-6 score	-6.353	2.865	-0.230	-2.217	-12.070, -0.635	0.030*	-13.064, -0.858	0.043*
Serum HDL-c (mg/dL)	-0.034	0.172	-0.022	-0.201	-0.377, 0.308	0.842	-0.328, 0.214	0.831
Mid-puberty (Tanner III)	-11.664	3.850	-0.355	-3.030	-19.345, -3.981	0.003*	-20.000, -3.895	0.005*
Late puberty (Tanner IV)	-10.588	4.185	-0.299	-2.530	-18.940, -2.237	0.014*	-19.679, -2.865	0.021*

Females (n= 44):

Predictor	B	SE	β	t	OLS 95% CI	*p* (OLS)	BCa 95% CI	*p* (BCa)
BMI z-score	6.080	2.073	0.431	2.933	1.883, 10.276	0.006*	2.072, 10.950	0.005*
ACQ-6 score	-0.137	2.070	-0.009	-0.066	-4.328, 4.054	0.948	-3.557, 5.914	0.926
Serum HDL-c (mg/dL)	0.475	0.213	0.341	2.227	0.043, 0.907	0.032*	0.050, 0.988	0.052
Mid-puberty (Tanner III)	-10.880	5.030	-0.365	-2.163	-21.063, -0.697	0.037*	-21.452, -2.521	0.031*
Late puberty (Tanner IV)	-13.525	4.386	-0.504	-3.084	-22.404, -4.647	0.004*	-21.559, -5.076	0.006*

[a] For the male BMI z-score coefficient, the bootstrap BCa 95% CI contains zero despite *p* BCa = 0.004, whereas the OLS 95% CI does not contain zero and is consistent with *p* OLS = 0.007. This discrepancy reflects the bootstrap distribution skewness (bias = -0.242; SE boot = 0.771), attributable to a single influential observation (Cook's D = 3.008). After exclusion of this observation in the sensitivity analysis (Table S2), the BMI z-score effect attenuated to β = -0.023 ($p = 0.839$; n = 73), confirming that the primary model coefficient was driven by a single observation rather than a robust association. See Section 3.3.2 and footnote [c] of Table 4 for a full discussion. * $p < 0.05$. Abbreviations: ACQ-6: Asthma Control Questionnaire 6-item; BCa: bias-corrected and accelerated bootstrap; BMI: body mass index; CI: confidence interval; FVC: forced vital capacity; HDL-c: high-density lipoprotein cholesterol; OLS: ordinary least squares; SE: standard error.

Table S6. Multiple-Testing Correction (Benjamini–Hochberg FDR) for Sex-Stratified Bootstrap BCa *p*-Values across the Three Pulmonary Function Outcomes.

The 30 sex-stratified contrasts (5 predictors × 3 outcomes × 2 sexes) reported in Tables 3, 4, and 5 were subjected to Benjamini-Hochberg false discovery rate (FDR) control, with adjusted p-values (q-values) calculated as q = min[1, p × m / rank] and adjusted to be monotonic in rank (m = 30).

Outcome	Sex	Predictor	Raw p (BCa)	BH rank	Adjusted q	*q < 0.05*	*q < 0.10*
FEV1%	Males	BMI z-score	0.079	17	0.139		
FEV1%	Males	ACQ-6	0.110	18	0.183		
FEV1%	Males	HDL-c	0.519	26	0.599		
FEV1%	Males	Mid- puberty (Tanner III)	0.002	2	0.020	*	*
FEV1%	Males	Late puberty (Tanner IV)	0.001	2	0.020	*	*
FEV1%	Females	BMI z-score	0.006	7	0.020	*	*
FEV1%	Females	ACQ-6	0.020	11	0.052		*
FEV1%	Females	HDL-c	0.003	3	0.020	*	*
FEV1%	Females	Mid- puberty (Tanner III)	0.127	19	0.201		
FEV1%	Females	Late puberty (Tanner IV)	0.306	23	0.399		
FVC%	Males	BMI z-score	0.004	4	0.020	*	*
FVC%	Males	ACQ-6	0.043	15	0.086		*

FVC%	Males	HDL-c	0.831	29	0.860		
FVC%	Males	Mid- puberty (Tanner III)	0.005	5	0.020	*	*
FVC%	Males	Late puberty (Tanner IV)	0.021	12	0.052		*
FVC%	Females	BMI z-score	0.005	6	0.020	*	*
FVC%	Females	ACQ-6	0.926	30	0.926		
FVC%	Females	HDL-c	0.052	16	0.097		*
FVC%	Females	Mid- puberty (Tanner III)	0.031	14	0.066		*
FVC%	Females	Late puberty (Tanner IV)	0.006	8	0.020	*	*
FEV1/FVC%	Males	BMI z-score	0.015	10	0.045	*	*
FEV1/FVC%	Males	ACQ-6	0.679	27	0.736		
FEV1/FVC%	Males	HDL-c	0.687	28	0.736		
FEV1/FVC%	Males	Mid- puberty (Tanner III)	0.439	25	0.527		
FEV1/FVC%	Males	Late puberty (Tanner IV)	0.149	20	0.214		
FEV1/FVC%	Females	BMI z-score	0.157	22	0.214		
FEV1/FVC%	Females	ACQ-6	0.006	9	0.020	*	*
FEV1/FVC%	Females	HDL-c	0.028	13	0.065		*
FEV1/FVC%	Females	Mid- puberty (Tanner III)	0.412	24	0.515		
FEV1/FVC%	Females	Late puberty (Tanner IV)	0.152	21	0.214		

[a]Sensitivity analysis (Table S2) showed that this association was driven by a single influential observation (Cook's D = 3.008); see also footnote [c] in main Table 4.

Of the 17 contrasts with raw $p < 0.05$, 9 retained statistical significance after FDR correction at $q < 0.05$ (pubertal stage effects on FEV1% and FVC% in both sexes; HDL-c and BMI z-score in FEV1% females; BMI z-score in FEV1/FVC% males and ACQ-6 in FEV1/FVC% females). Six additional contrasts fell into the q < 0.10 zone, including the effect of HDL-c on FEV1/FVC% in females ($q = 0.065$), which was one of the main findings of this study. Under stringent FDR control ($q < 0.05$), the female-specific HDL-c association with airflow obstruction did not survive correction and should, therefore, be regarded as a hypothesis-generating preliminary finding rather than a confirmed association. The dominant pubertal-stage effects on volumetric pulmonary function remained robust across both sexes after FDR correction.

* *p* or *q* below the threshold.
